# Supplementary material for: Helianthus annuus Seed Extract Affects Weight and Body Composition of Healthy Obese Adults during 12 Weeks of Consumption: A Randomized, Double-Blind, Placebo-Controlled Pilot Study
Source: Nutrients. 2019 May 15;11(5):1080. doi: 10.3390/nu11051080 (PMC6566515; doi:10.3390/nu11051080)
Supplement: Supplementary file 1 [file nutrients-11-01080-s001.pdf]

Supplementary Materials

Helianthus annuus seed extract affects weight and body composition of healthy obese adults during 12 weeks of consumption: A randomized, double-blind, placebo-controlled pilot study

Aurélie Leverrier [1], David Daguet [1,*], Wim Calame [2], Pierre Dhoye [1] and Shyam Prasad Kodimule [1,3]

[1] Vidya Europe SAS, 7 avenue de Norvège, 91140 Villebon sur Yvette, France; aurelie@vidyaeurope.eu (A.L.); pierre@vidyaeurope.eu (P.D.); shyamprasad@vidyaherbs.com (S.P.K.)
[2] StatistiCal BV, Strandwal 148, 2241 MN Wassenaar, The Netherlands; w.calame@kpnplanet.nl
[3] R&D Centre for Excellence, Vidya Herbs Pvt. Ltd, #14A, Jigani I Phase, Bangalore 560105, Karnataka, India
* Correspondence: david@vidyaeurope.eu; Tel.: +91-33-2-18-56-24-51

Table of content **Pages**

Table S1. Summary of the study follow-up intervention — Page S - 2

Table S2. Means of change values ± SD per parameter and per visit compared to baseline for all the subjects — Page S – 3

Table S3. Means of change values ± SD per parameter and per visit compared to baseline for subjects above 30 years — Page S - 4

Table S4. Means of change values ± SD per parameter and per visit compared to baseline for women above 30 years — Page S - 5

Table S1. Summary of the study follow-up intervention

	V0	V1	V2	V3	V4	V5
Informed Consent Signature	x					
Clinical History	x					
Vital Signs	x		x	x	x	x
Weight, Height, BMI	x		x	x	x	x
Waist circumference	x		x	x	x	x
ECG	x					x
Bioelectric impedance analysis	x		x	x	x	x
Analytical examination	x		x	x	x	x
Randomization		x				
Treatment delivery		x	x	x	x	
Treatment return			x	x	x	x
IPAQ		x				x
Hypocaloric diet		x				
24h reminder			x	x	x	x
Adverse events			x	x	x	x
Concomitant medication			x	x	x	x

Table S2. Means of change values ± SD per parameter and per visit compared to baseline for all the subjects

	Placebo (N = 20)					Sunflower extract (N = 30)				
	Week 3 (N = 19)	Week 6 (N = 18)	Week 9 (N = 18)	Week 12 (N = 18)	p-value (V5 -V0)	Week 3 (N = 29)	Week 6 (N = 28)	Week 9 (N = 28)	Week 12 (N = 28)	p-value (V5 -V0)
Weight (kg)	-2.33 ± 2.45	-4.07 ± 2.89	-4.85 ± 3.81	-5.53 ± 4.13	0.000	-2.89 ± 2.16	-4.83 ± 2.76	-6.11 ± 3.78	-6.90 ± 4.45	0.000
BMI (kg/m^2)	-0.76 ± 0.89	-1.38 ± 1.02	-1.66 ± 1.33	-1.88 ± 1.34	0.000	-0.98 ± 0.73	-1.68 ± 0.87	-2.14 ± 1.20	-2.60 ± 1.72	0.000
WC (cm)	-0.53 ± 5.84	-1.50 ± 6.09	-3.78 ± 6.49	-4.75 ± 7.48	0.000	-3.36 ± 2.33	-4.93 ± 3.16	-6.18 ± 2.90	-8.44 ± 4.28	0.000
MM (kg)	-0.75 ± 1.39	-1.06 ± 1.21	-1.14 ± 1.78	-1.54 ± 1.83	0.000	-1.03 ± 1.44	-1.23 ± 1.61	-1.77 ± 1.58	-1.88 ± 1.63	0.000
FM (kg)	-1.58 ± 1.84	-3.01 ± 2.03	-3.71 ± 2.53	-3.98 ± 3.01	0.000	-1.60 ± 1.88	-3.49 ± 2.35	-4.22 ± 3.17	-4.87 ± 3.63	0.000
MM/FM (ratio)	0.07 ± 0.14	0.12 ± 0.15	0.17 ± 0.22	0.19 ± 0.27	0.000	0.04 ± 0.10	0.13 ± 0.14	0.16 ± 0.19	0.20 ± 0.22	0.000
TBW (kg)	-0.54 ± 1.02	-0.82 ± 0.90	-0.79 ± 1.59	-1.13 ± 1.35	0.000	-0.30 ± 2.47	-0.30 ± 2.32	-0.84 ± 2.39	-0.90 ± 2.76	0.000
Fat (%)	-0.71 ± 1.23	-1.42 ± 1.15	-1.89 ± 1.57	-2.03 ± 1.99	0.000	-0.64 ± 1.28	-1.77 ± 1.62	-2.05 ± 1.95	-2.44 ± 2.13	0.000
Glucose (mg/dl) [a]	-4.79 ± 7.10	-6.06 ± 11.03	-3.28 ± 9.57	-2.61 ± 11.07	0.561	-3.14 ± 13.55	-2.64 ± 13.06	-2.79 ± 11.47	-3.71 ± 9.53	0.054
HbA1c (%) [b]	-0.15 ± 0.17	-0.14 ± 0.12	-0.15 ± 0.13	-0.13 ± 0.13	0.001	-0.12 ± 0.22	-0.17 ± 0.29	-0.20 ± 0.32	-0.13 ± 0.28	0.002
Cholesterol (mg/dL)	-26.63 ± 19.37	-19.94 ± 23.46	-15.39 ± 25.36	-8.72 ± 23.14	0.572	-24.52 ± 26.56	-23.25 ± 28.90	-15.00 ± 25.94	-18.43 ± 25.73	0.019
TG (mg/dL)	-18.84 ± 58.61	-15.22 ± 53.09	-15.89 ± 55.52	-23.94 ± 61.31	0.061	-24.31 ± 43.18	-23.54 ± 57.16	-9.36 ± 55.09	-25.46 ± 48.77	0.077
LDL (mg/dL) [c]	-15.72 ± 15.58	-10.94 ± 17.51	-6.71 ± 18.18	-3.53 ± 18.02	0.930	-15.57 ± 24.13	-13.15 ± 28.42	-9.58 ± 24.82	-9.69 ± 20.51	0.277
HDL (mg/dL)	-7.74 ± 9.31	-6.11 ± 8.26	-4.67 ± 8.01	-0.78 ± 9.14	0.965	-3.90 ± 5.04	-5.04 ± 5.63	-2.82 ± 5.44	-2.75 ± 4.50	0.055
LDL/HDL (ratio) [c]	0.07 ± 0.49	0.11 ± 0.42	0.11 ± 0.59	-0.03 ± 0.50	0.946	-0.10 ± 0.50	0.02 ± 0.45	-0.03 ± 0.51	-0.02 ± 0.50	0.905
FFA (nmol/dL) [d]	0.22 ± 0.24	0.15 ± 0.22	0.09 ± 0.22	0.14 ± 0.25	0.178	0.19 ± 0.19	0.15 ± 0.26	0.09 ± 0.20	0.09 ± 0.24	0.358

Some individual data were missing and consequently the number of subjects included in the statistical analysis changed for some parameters:

[a] Blood glucose: 19,18,18 and 17 subjects analysed at week 3, 6, 9 and 12, respectively, for placebo against 26,27,27 and 28, respectively, for sunflower extract

[b] HbA1c: 18, 17, 17 and 16 subjects analysed at week 3, 6, 9 and 12, respectively, for placebo against 25,27,27,27, respectively, for sunflower extract

[c] For LDL, and consequently LDL/HDL ratio, the number of analysed subjects were 18 at week 3 and then 17 at weeks 6-12, for placebo, and 28 at week 3 and the 26 at weeks 6-12, for sunflower extract

[d] FFA: 18 subjects analysed for all weeks for placebo, against 26, 27, 28 and 28 subjects at week 3, 6, 9 and 12 respectively for sunflower extract

Table S3. Means of change values ± SD per parameter and per visit compared to baseline for subjects above 30 years

	Placebo (N = 18)					Sunflower extract (N = 28)				
	Week 3 (N = 17)	Week 6 (N = 17)	Week 9 (N = 17)	Week 12 (N = 17)	p-value (V5 -V0)	Week 3 (N = 27)	Week 6 (N = 26)	Week 9 (N = 26)	Week 12 (N = 26)	p-value (V5 -V0)
Weight (kg)	-2.81 ± 1.94	-4.21 ± 2.91	-5.06 ± 3.82	-5.71 ± 4.19	0.000	-3.09 ± 2.08	-5.10 ± 2.67	-6.50 ± 3.64	-7.28 ± 4.39	0.000
BMI (kg/m²)	-0.92 ± 0.72	-1.42 ± 1.03	-1.72 ± 1.34	-1.94 ± 1.36	0.000	-1.04 ± 0.71	-1.78 ± 0.83	-2.27 ± 1.13	-2.75 ± 1.70	0.000
WC (cm)	-0.47 ± 6.19	-1.53 ± 6.28	-3.94 ± 6.65	-4.91 ± 7.68	0.000	-3.41 ± 2.39	-5.00 ± 3.22	-6.39 ± 2.82	-8.82 ± 4.14	0.000
MM (kg)	-0.86 ± 1.43	-1.20 ± 1.09	-1.15 ± 1.83	-1.69 ± 1.77	0.000	-1.10 ± 1.47	-1.20 ± 1.66	-1.75 ± 1.59	-1.86 ± 1.65	0.000
FM (kg)	-1.95 ± 1.40	-3.01 ± 2.09	-3.91 ± 2.47	-4.02 ± 3.10	0.000	-1.72 ± 1.89	-3.77 ± 2.18	-4.62 ± 2.91	-5.26 ± 3.46	0.000
MM/FM (ratio)	0.08 ± 0.14	0.11 ± 0.15	0.18 ± 0.23	0.19 ± 0.28	0.000	0.05 ± 0.10	0.15 ± 0.14	0.18 ± 0.18	0.22 ± 0.21	0.000
TBW (kg)	-0.63 ± 1.04	-0.93 ± 0.80	-0.80 ± 1.64	-1.24 ± 1.31	0.000	-0.30 ± 2.57	-0.23 ± 2.39	-0.78 ± 2.46	-0.85 ± 2.85	0.003
Fat (%)	-0.87 ± 1.14	-1.36 ± 1.16	-2.01 ± 1.54	-2.00 ± 2.04	0.000	-0.70 ± 1.32	-1.96 ± 1.51	-2.31 ± 1.76	-2.69 ± 1.97	0.000
Glucose (mg/dl) [a]	-5.50 ± 7.53	-6.06 ± 11.37	-3.18 ± 9.85	-2.41 ± 11.37	0.722	-3.73 ± 13.70	-3.50 ± 13.00	-3.38 ± 11.67	-4.35 ± 9.58	0.031
HbA1c (%) [b]	-0.18 ± 0.16	-0.14 ± 0.12	-0.16 ± 0.13	-0.12 ± 0.13	0.003	-0.12 ± 0.21	-0.18 ± 0.28	-0.22 ± 0.32	-0.14 ± 0.29	0.002
Cholesterol (mg/dL)	-28.18 ± 19.94	-20.76 ± 23.91	-16.41 ± 25.76	-8.65 ± 23.85	0.616	-25.59 ± 27.22	-24.04 ± 29.79	-15.96 ± 26.68	-18.31 ± 25.86	0.029
TG (mg/dL)	-26.41 ± 54.81	-16.18 ± 54.56	-18.88 ± 55.71	-23.29 ± 63.13	0.070	-26.26 ± 44.00	-25.19 ± 59.06	-10.00 ± 57.08	-26.08 ± 50.20	0.102
LDL (mg/dL) [c]	-16.19 ± 16.03	-11.50 ± 17.92	-7.25 ± 18.63	-3.69 ± 18.60	0.914	-16.38 ± 24.84	-13.63 ± 29.51	-10.50 ± 25.63	-9.71 ± 20.51	0.314
HDL (mg/dL) [c]	-7.41 ± 9.82	-6.18 ± 8.51	-4.53 ± 8.23	-0.71 ± 9.42	0.804	-3.78 ± 5.06	-4.88 ± 5.70	-2.68 ± 5.72	-2.42 ± 4.47	0.116
LDL/HDL (ratio) [c]	0.04 ± 0.50	0.10 ± 0.43	0.09 ± 0.60	-0.04 ± 0.52	0.890	-0.12 ± 0.51	0.00 ± 0.46	-0.05 ± 0.52	-0.03 ± 0.50	0.983
FFA (nmol/dL) [d]	0.20 ± 0.25	0.15 ± 0.23	0.09 ± 0.22	0.15 ± 0.26	0.159	0.19 ± 0.20	0.16 ± 0.27	0.09 ± 0.20	0.07 ± 0.22	0.605

Some individual data were missing and consequently the number of subjects included in the statistical analysis changed for some parameters:

[a] Blood glucose: 16 subjects were analysed at week 3 and 17 subjects for the 3 remaining visits, for placebo, against 26 subjects analysed for all the visit for sunflower extract

[b] HbA1c: 15,16,16, and 15 subjects analysed at week 3, 6, 9 and 12, respectively, for placebo against 25, 26, 26 and 26 subjects, respectively, for sunflower extract

[c] For LDL: 16 subjects were analysed at all weeks for placebo, against 26 subjects at week 3 and then 24 subjects at week 6-12 for sunflower extract. For HDL: 17 subjects were analysed at all weeks for placebo, against 27 subjects at week 3, 25 at weeks 6 and 9, and 26 at week 12 for sunflower extract. For LDL/HDL ratio: 16 subjects analysed for placebo for all the visits, against 26 at week 3 and then 24 at weeks 6-12 for sunflower extract

[d] FFA: 16 subjects analysed at week 3 and then 17 for the remaining visits, for placebo, against 24, 25, 26 and 26 subjects at week 3, 6, 9 and 12, respectively, for sunflower extract

Table S4. Means of change values ± SD per parameter and per visit compared to baseline for women above 30 years

	Placebo (N = 14)					Sunflower extract (N = 16)				
	Week 3 (N = 13)	Week 6 (N = 13)	Week 9 (N = 13)	Week 12 (N = 13)	p-value (V5 -V0)	Week 3 (N = 16)	Week 6 (N = 15)	Week 9 (N = 15)	Week 12 (N = 15)	p-value (V5 -V0)
Weight (kg)	-2.72 ± 1.63	-4.00 ± 2.38	-4.72 ± 2.83	-4.93 ± 2.45	0.000	-2.60 ± 1.97	-4.21 ± 2. 18	-5.40 ± 2.94	-6.01 ± 3.63	0.000
BMI (kg/m^2)	-0.98 ± 0.60	-1.46 ± 0.87	-1.74 ± 1.05	-1.81 ± 0.90	0.000	-1.00 ± 0.71	-1.59 ± 0.81	-2.07 ± 1.09	-2.31 ± 1.36	0.000
WC (cm)	0.27 ± 6.83	-0.58 ± 6.65	-2.58 ± 6.77	-3.23 ± 7.62	0.002	-3.63 ± 2.66	-5.30 ± 3.22	-6.41 ± 2.21	-9.15 ± 3.38	0.000
MM (kg)	-0.85 ± 1.07	-1.20 ± 1.12	-1.32 ± 1.60	-1.37 ± 1.53	0.000	-0.56 ± 1.20	-0.70 ± 1.48	-1.25 ± 1.23	-1.22 ± 1.44	0.000
FM (kg)	-1.88 ± 0.96	-2.80 ± 1.55	-3.40 ± 1.74	-3.56 ± 1.60	0.000	-2.04 ± 1.45	-3.51 ± 1.52	-4.15 ± 2.30	-4.72 ± 2.86	0.000
MM/FM (ratio)	0.04 ± 0.04	0.06 ± 0.04	0.08 ± 0.06	0.09 ± 0.06	0.000	0.06 ± 0.08	0.11 ± 0.08	0.11 ± 0.09	0.14 ± 0.11	0.000
TBW (kg)	-0.62 ± 0.79	-0.95 ± 0.83	-0.96 ± 1.20	-1.00 ± 1.14	0.000	-0.39 ± 0.88	-0.29 ± 0.73	-0.89 ± 0.88	-0.89 ± 1.05	0.000
Fat (%)	-0.75 ± 0.67	-1.12 ± 0.77	-1.46 ± 1.02	-1.62 ± 1.11	0.000	-1.06 ± 1.18	-1.89 ± 1.32	-2.05 ± 1.43	-2.41 ± 1.71	0.000
Glucose (mg/dl)	-5.54 ± 8.43	-3.69 ± 8.59	-2.54 ± 9.90	-5.00 ± 6.42	0.240	0.56 ± 8.61	0.47 ± 6.65	0.80 ± 6.91	-0.53 ± 7.06	0.640
HbA1c (%) [a]	-0.16 ± 0.20	-0.15 ± 0.13	-0.15 ± 0.14	-0.11 ± 0.14	0.037	-0.11 ± 0.23	-0.16 ± 0.25	-0.09 ± 0.17	-0.07 ± 0.16	0.299
Cholesterol (mg/dL)	-28.46 ± 19.60	-18.15 ± 25.43	-11.00 ± 24.73	-4.85 ± 21.51	0.715	-21.13 ± 29.76	-20.40 ± 25.80	-10.80 ± 25.88	-15.53 ± 18.45	0.209
TG (mg/dL)	-12.46 ± 24.62	-0.08 ± 33.21	-2.38 ± 23.57	-6.54 ± 30.75	0.762	-15.94 ± 37.12	-20.60 ± 39.77	-1.80 ± 51.39	-25.00 ± 28.37	0.120
LDL (mg/dL)	-16.15 ± 15.57	-10.54 ± 19.01	-4.69 ± 19.85	-2.00 ± 17.69	0.572	-13.56 ± 28.84	-10.53 ± 23.91	-7.33 ± 26.02	-8.20 ± 17.88	0.512
HDL (mg/dL)	-9.85 ± 9.96	-7.77 ± 8.93	-6.00 ± 8.67	-1.69 ± 10.44	0.951	-4.31 ± 5.78	-5.60 ± 6.19	-3.00 ± 7.01	-2.27 ± 5.50	0.431
LDL/HDL (ratio)	0.14 ± 0.45	0.19 ± 0.43	0.24 ± 0.54	0.06 ± 0.49	0.490	-0.03 ± 0.60	0.13 ± 0.33	0.05 ± 0.50	0.00 ± 0.38	0.752
FFA (nmol/dL) [b]	0.21 ± 0.25	0.13 ± 0.24	0.08 ± 0.24	0.15 ± 0.28	0.206	0.21 ± 0.23	0.16 ± 0.26	0.05 ± 0.22	0.04 ± 0.20	0.753

Some individual data were missing and consequently the number of subjects included in the statistical analysis changed for some parameters:

[a] HbA1c: 12 subjects were analysed at all the visits, for placebo, against 15 subjects for all the visit for sunflower extract

[b] FFA: 12 subjects analysed at week 3 and then 13 subjects at the remaining visits, for placebo, against 13, 14, 15 and 15 subjects at week3, 6, 9 and 12, respectively for sunflower extract
